# Percentage of intrathoracic stomach predicts operative and post-operative morbidity, persistent reflux and PPI requirement following laparoscopic hiatus hernia repair and fundoplication

**DOI:** 10.1007/s00464-022-09701-0

**Published:** 2022-10-24

**Authors:** A. M. Cocco, V. Chai, M. Read, S. Ward, M. A. Johnson, L. Chong, C. Gillespie, M. W. Hii

**Affiliations:** 1 The Department of Surgery, The University of Melbourne, St Vincent’s Hospital Melbourne, Melbourne, Australia; 2 Upper GI and Hepatobiliary Surgical Unit, St Vincent’s Hospital Melbourne, Melbourne, Australia; 3 Upper GI and Hepatobiliary Surgical Unit, Eastern Health, Melbourne, Australia; 4 Upper GI and Hepatobiliary Surgical Unit, The Royal Melbourne Hospital, Melbourne, Australia

**Keywords:** Hiatus hernia repair, Fundoplication, Oesophageal, Laparoscopic, Reflux, Morbidity

## Abstract

**Purpose:**

Large hiatus hernias are relatively common and can be associated with adverse symptoms and serious complications. Operative repair is indicated in this patient group for symptom management and the prevention of morbidity. This study aimed to identify predictors of poor outcomes following laparoscopic hiatus hernia repair and fundoplication (LHHRaF) to aid in counselling potential surgical candidates.

**Methodology:**

A retrospective analysis was performed from a prospectively maintained, multicentre database of patients who underwent LHHRaF between 2014 and 2020. Revision procedures were excluded. Hernia size was defined as the intraoperative percentage of intrathoracic stomach, estimated by the surgeon to the nearest 10%. Predictors of outcomes were determined using a prespecified multivariate logistic regression model.

**Results:**

625 patients underwent LHHRaF between 2014 and 2020 with 443 patients included. Median age was 65 years, 62.9% were female and 42.7% of patients had ≥ 50% intrathoracic stomach. In a multivariate regression model, intrathoracic stomach percentage was predictive of operative complications (*P* = 0.014, OR 1.05), post-operative complications (*P* = 0.026, OR 1.01) and higher comprehensive complication index score (*P* = 0.023, OR 1.04). At 12 months it was predictive of failure to improve symptomatic reflux (*P* = 0.008, OR 1.02) and persistent PPI requirement (*P* = 0.047, OR 1.02). Operative duration and blood loss were predicted by BMI (*P* = 0.004 and < 0.001), Type III/IV hernias (*P* = 0.045 and *P* = 0.005) and intrathoracic stomach percentage (*P* = 0.009 and *P* < 0.001). Post-operative length of stay was predicted by age (*P* < 0.001) and emergency presentation (*P* = 0.003).

**Conclusion:**

In a multivariate regression model, intrathoracic stomach percentage was predictive of operative and post-operative morbidity, PPI use, and failure to improve reflux symptoms at 12 months.

Hiatus hernias are a common condition and are defined as the extension of normally intra-abdominal viscera, most commonly the stomach, through the oesophageal hiatus of the diaphragm into the posterior mediastinum [1]. The prevalence of hiatal hernias is difficult to assess due to the absence of large-scale population-based studies; however, estimates range between 13 and 59% [2]. Age and obesity are associated with increased risk of developing a hiatus hernia [3, 4] with a recent meta-analysis finding an odds ratio for hiatus hernia of 1.93 and 2.17 for people with a BMI more than 25 or age greater than 50, respectively [5].

Many patients with hiatus hernia are asymptomatic or have only mild symptoms, most commonly heartburn and regurgitation [6]. Larger hernias are more likely to be associated with severe symptoms due to lower oesophageal sphincter incompetence [7] and may also have symptoms due to mass effect and obstruction, including dysphagia, chest pain, exertional dyspnoea, vomiting and anaemia [8].

The assessment of hiatal hernia size is challenging and can be defined in terms of absolute or relative size. Absolute size can be measured in terms of cranio-caudal hernia length, endoscopically or radiologically, or the size of the diaphragmatic defect, measured by the vertical distance between the crus and apex and the horizontal distance between the crural pillars. Relative size can be determined according to degree of intrathoracic stomach.

Large hiatus hernias have been defined as > 5 cm in length, as assessed on high-resolution manometry [9] and by this definition have been found to be associated with greater reflux symptoms [9] and severity of esophagitis [10]. Proportion of intrathoracic stomach has been previously measured preoperatively with barium oesophagram [11] or CT imaging. Finally, the size of the hiatal hernial defect itself can be measured intraoperatively. This was found to have no association with quality of life (QoL) or pre-operative symptoms [12] but increased likelihood of recurrence. Giant hiatus hernia have been defined variably within the literature including as all type III and IV hernias or by proportion of intrathoracic stomach, ranging from 30 to 50% [11, 13].

Operative repair in patients with hiatus hernia is indicated in symptomatic patients and in the small subgroup that present emergently with obstruction or volvulus [1]. Current guidelines suggest that repair of a type I hernia in the absence of reflux disease is not necessary [1] but that surveillance or repair can be considered for type II–IV hernias due to a higher risk of progression to symptoms [14] and higher rates of serious morbidity [15]. High perioperative mortality rates up to 22% have been reported following emergency repair of paraesophageal hernias [16–18] and is a consideration in the decision to repair earlier, especially with the elderly [19].

Laparoscopic hiatal hernia repair and fundoplication (LHHRaF) is a technically challenging procedure. Reported rates of total post-operative morbidity from high-volume centres ranges from 10 to 26% [20–25]. The most common post-operative complications are cardiorespiratory compromise, nausea and vomiting, dysphagia, perforation and anatomical recurrence [1, 25]. Morbidity and mortality have been primarily associated with urgency [24], age and comorbidity [15]. It has also been found that open and transthoracic repair are associated with higher mortality, overall morbidity and severe morbidity [26] and, as such, laparoscopic repair is now the standard of care.

Most patients who undergo LHHRaF derive great benefit, with 73–90% achieving symptom resolution over the long term [18, 27]. With LHHRaF presenting a safe elective surgical option with favourable outcomes, it is important to identify methods to stratify risk for patients. The primary aim of this analysis was to perform a multivariate analysis to determine predictors of poor outcome following laparoscopic hiatus hernia repair and fundoplication.

## Methods

A retrospective analysis was conducted on a prospectively maintained multicentre database of adult patients undergoing laparoscopic hiatus hernia repair and fundoplication between 2014 and 2020. Study data were collected and managed using (REDCap) Research Electronic Data Capture data capture tools [28, 29] hosted at The University of Melbourne. Six centres in Melbourne, Australia participated, including three public tertiary referral centres and three private centres. All procedures were performed by one of six subspecialist oesophagogastric surgeons.

The study included all patients undergoing laparoscopic hiatus hernia repair with fundoplication. Revision procedures and those without intraoperative measurement of intrathoracic stomach percentage were excluded from this study. Patient demographics, indications for surgery, operative and post-operative metrics were recorded.

It has been postulated that recurrence of large hiatus hernias may, in part, be due to shortening of the oesophagus with tension on the gastro-oesophageal junction retracting the stomach back into the thorax [30]. Oesophageal shortening is linked to erosive oesophagitis and stricturing disease, although the incidence of this has markedly reduced since the adoption of proton pump inhibitor therapy [31]. Hence, oesophageal lengthening via a Collis gastroplasty has been proposed to reduce recurrence risk. Whilst Collis gastroplasty potentially reduces radiological hernia recurrence rates, rates of symptoms recurrence or re-operation may not improve. In addition, there may be an increase in perioperative complications with a small risk of gastric perforation, which itself carries a high burden of morbidity and mortality [32]. The neoesophagus formed by the gastroplasty also continues to secrete acid proximal to an intact fundoplication and can result in mucosal damage [30, 33]. It also does not exhibit the same peristaltic activity as the native oesophagus, introducing higher rates of post-operative dysphagia [34]. Oesophageal lengthening in hiatus hernia repair has not been universally adopted [35] and rates of utilisation in published series vary between 0 and more than 50% [36, 37]. In published Australasian series, lower rates are typically seen [38]. For these reasons, our technique incorporates discretionary mesh and/or pledget use and a high mediastinal dissection if required, but not Collis gastroplasty.

Hernia size was defined as the intrathoracic stomach percentage, estimated intraoperatively by the operative surgeon to the nearest 10%. The level of the incisura angularis was taken as representing a point midway along the stomach (i.e. 50%). The operative technique prior to intraoperative estimation of intrathoracic stomach percentage was standardised with regards to pneumoperitoneum pressure, muscle relaxant general anaesthesia and gastric decompression.

Estimations were performed using endoscopy, laparoscopy, radiology and manometry where available to assess for correlation. Manometry was reserved for patients with a suspected anatomical abnormality, such as oesophageal dilation on endoscopy or barium swallow or CT, or patients with symptomatic dysphagia. Manometry is less reliable in very large hiatus hernias with frequent coiling within the hernia, failure to traverse the diaphragm and altered oesophageal pressure topography measurements [39]. Estimation was based on the gastric volume or cross-sectional area in the coronal plane within the thorax, again with the level of the incisura angularis taken as representing a point midway along the stomach (i.e. 50%). Prior to study commencement all surgeons assessed both pre-operative and operative imaging to generate consensus and reduce interobserver variability. Rounding the value to the nearest 10% was also introduced to reduce variation.

Primary analysis was performed comparing variables against mortality, morbidity and clinical measures of hiatal repair efficacy. These included reliance on proton pump inhibitors, self-reported symptoms using a modified DeMeester questionnaire (DeMeester Symptom Scores) and clinical, endoscopic and radiological assessment of recurrence (where available). Patients continuing PPI therapy for non-reflux indications were excluded from PPI-related outcomes. These were reassessed at 12- and 24-month follow-up. Age, sex, BMI, comorbidity [as assessed by American Society of Anaesthesiologists (ASA) score and Charlson Comorbidity Index (CCI)] and anatomical factors of hernia size and type were included in the analysis. Complications were measured and graded using Clavien–Dindo complication grades [40]. Operative complications were defined as intra-abdominal bleeding, splenic or gastric injury, perforation or pneumothorax. Severe complications were defined as a Clavien–Dindo score ≥ 3a.

Multivariate models were prespecified using clinically associated variables (age, gender, BMI, hernia size, hernia type, CCI excluding age, strangulation, emergency presentation and intraoperative mesh use) to guard against spurious findings.

Statistical analysis was completed using IBM Statistical Package for Social Sciences (SPSS) version 22 (IBM Corp, Armonk NY). Descriptive statistics were reported as absolute numbers and percentage for binary and ordinal variables, whilst continuous variables were described using median and interquartile range or mean and standard deviation. Denominators were adjusted for incomplete or missing data. Univariate statistical analysis was completed using Pearson’s Chi-square analysis or independent sample *T* test. Multivariate analysis was completed using enter/simultaneous method logistic regression analysis for binary outcomes and linear regression analysis for continuous outcomes. Measures of significance were two-tailed.

## Surgical technique

Hiatus hernia repair is performed under general anaesthesia. Patients are placed in the lithotomy position. Four or five working laparoscopy ports and a Nathanson liver retractor (Cook Australia Ltd, Brisbane, Australia) are used. The two most superior short gastric vessels are divided with electrocautery and then circumferential mobilisation of the hiatus is performed. The hernia sac is mobilised and then dissected from the adjacent mediastinal structures. Where possible the accessory hepatic neurovascular bundle is preserved. Identification and preservation of the anterior and posterior vagal nerves are critical. Oesophageal mobilisation with high mediastinal dissection is performed such that the gastro-oesophageal junction can be reduced into the abdomen by 3 cm without tension. Posterior hiatal suture repair is preferred, with further anterior sutures placed at the discretion of the surgeon. Mesh reinforcement with pledgets, bioabsorbable mesh or permanent mesh is also performed at the discretion of the surgeon. All patients underwent a fundoplication procedure, usually a 270-degree posterior wrap. All patients had their proton pump inhibitor therapy ceased prior to discharge unless there were non-reflux indications for their use.

## Results

**Fig. 1:**
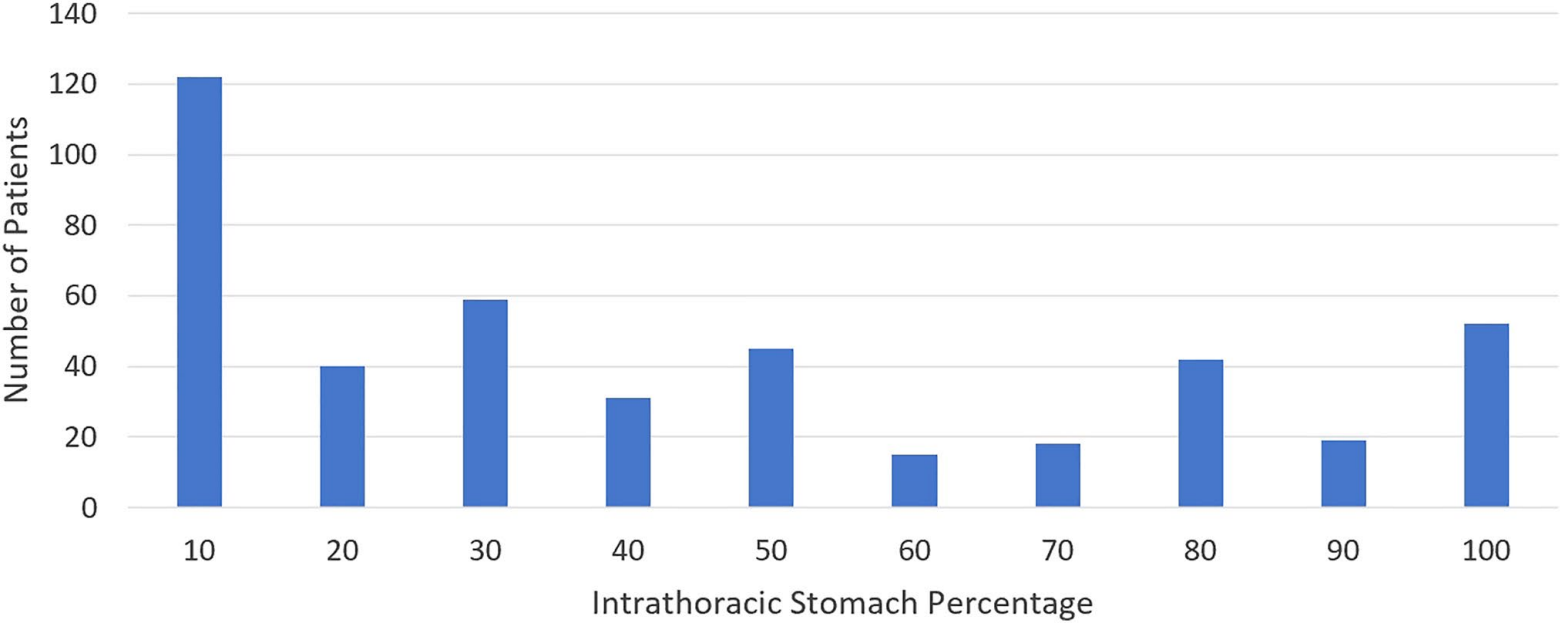
Frequency of intrathoracic stomach percentage (intraoperative assessment)

625 patients underwent laparoscopic hiatus hernia repair and/or fundoplication between 2014 and 2020. 72 patients had fundoplication only, 43 had revision procedures and 67 did not have intraoperative measurement of intrathoracic stomach percentage and were excluded. 443 patients were included in the study with 212 patients (47.8%) followed up at 12 months and 121 patients (27.3%) followed up at 24-month post-procedure. Demographic and baseline data are described in Table 1 and hernia size, as determined by intrathoracic stomach percentage, is shown in Fig. 1.

**Table 1:** Patient demographics

Total, *n*		443	BMI, median (IQR)		29.0 (26.4, 32.2)
Age, median (IQR)		65 (55, 73)	CCI, median (IQR)		2 (1, 3)
Gender, *n* (%)	Female	298 (62.9)	Type of hernia, *n* (%)	Type I	84 (20.0)
	Male	145 (37.1)		Type II	74 (17.5)
ASAc, *n* (%)	1	292 (66.4)		Type III	216 (51.2)
	2	148 (33.6)		Type IV	48 (11.4)
ASA, *n* (%)	I	21 (4.8)	Intrathoracic stomach percentage	Median (IQR)	30 (10–80)
	II	271 (61.6)			
	III	146 (33.2)	Admission type	Elective, n (%)	410 (93.0)
	IV	2 (0.4)		Emergency, n (%)	31 (7.0)

The most common symptoms indicating LHHRaF were heartburn (355 patients, 80%), regurgitation (285 patients, 64%), chest pain (207 patients, 46%) and dysphagia (183 patients, 41%). Patients presented with strangulation in 32 cases (7%).

Pre-operative endoscopy was performed in 94.4%, pH studies in 23.7% and manometry in 28.7%. 352 patients had pre-operative estimation of hernia size using endoscopy (77.7%), barium meal (15.6%), CT imaging (39.5%), manometry (9.3%) or laparoscopy (3.6%), with some estimations based on multiple modalities. Pre-operative hernia size estimations were significantly correlated with measurement intraoperatively (*R* = 0.9179, *P* < 0.001). 63.4% had no difference, 86.9% were within 10% and 93.5% were within 20%. 6.5% of cases had differences of more than 20% intrathoracic stomach between pre-operative estimation and intraoperative measurement. The majority of the discordant pre-operative estimates compared to intraoperative measurements were via endoscopy (90% of cases with more than 20% difference). Endoscopy also had the largest mean difference (7.2%). Laparoscopy was the most operatively concordant with mean difference of 1.7%.

**Fig. 2:**
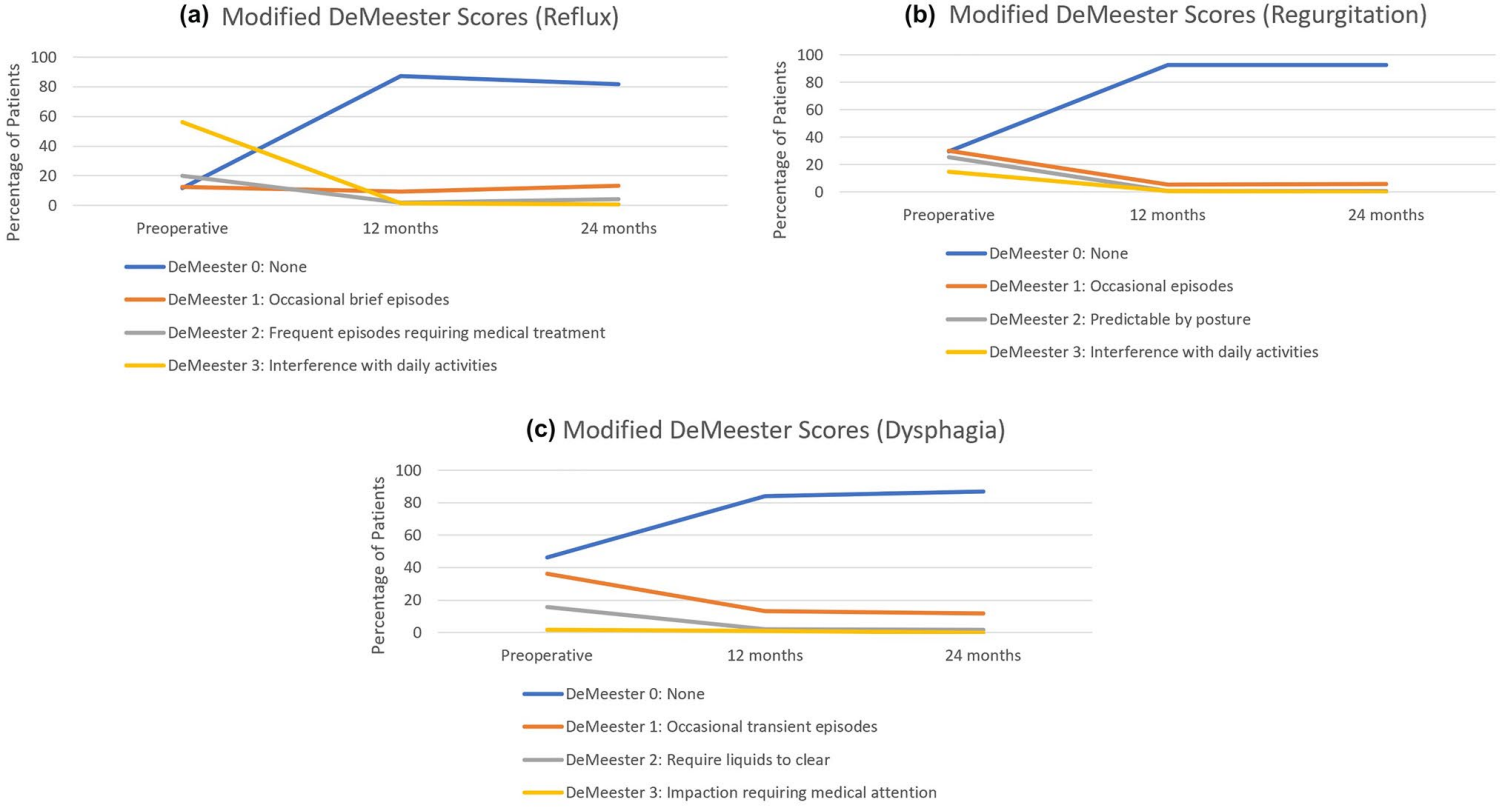
**a** Modified DeMeester symptom scores (Reflux). **b** Modified DeMeester symptom scores (Regurgitation). **c** Modified DeMeester symptom scores (Dysphagia)

Operative outcomes are shown in Table 2. Mesh and/or pledgets were used in 285 patients (64.3%). Most patients underwent a posterior 270-degree fundoplication (78%) with the remainder undergoing either an anterior 180-degree or posterior 180-degree fundoplication in approximately equal proportions. Rates of symptoms resolution, as assessed by Modified DeMeester Questionnaire (DeMeester Symptom Scores) at baseline and follow-up, are demonstrated in Fig. 2 and Table 3. There were no changes in operative technique or significant differences in outcomes when earlier cases in the series were compared to later cases. There was no significant difference in complication rates between surgeons (*P* = 0.278).

**Table 2:** Operative, post-operative and follow-up outcomes

*n*		433
Operative outcomes		
Operative duration (min)	Median (IQR)	115 (70–150)
Intraoperative blood loss (mL)	Median (IQR)	20 (10–30)
Operative complications	*n* (%)	14 (3.2)
Severe operative complications	*n* (%)	4 (0.9)
Post-operative outcomes		
Post-operative mortality (30-day)	*n* (%)	0 (0)
Post-operative complications	*n* (%)	74 (17.1)
Severe post-operative complications	*n* (%)	10 (2.3)
Post-operative length of stay (days)	Median (IQR)	2.0 (2.0–4.0)
Follow-up outcomes		
Post-operative time of follow-up	12 months	24 months
N	212	121
Anatomical recurrence, *n* (%)	5 (2.4)	4 (3.3)
Readmission, *n* (%)	3 (1.4)	1 (0.8)
PPI requirement, *n* (%)	40 (18.9)	28 (23.1)

**Table 3:** Mean modified DeMeester scores preoperatively and at follow-up

Modified DeMeester domain	Pre-operative	12-month post-operative	24-month post-operative	*P* value (pre-operative vs 12 months)	*P* value (pre-operative vs 24 months)
Reflux	2.21	0.18	0.24	< 0.001	< 0.001
Regurgitation	1.25	0.09	0.07	< 0.001	< 0.001
Dysphagia	0.73	0.20	0.15	< 0.001	< 0.001

Multivariate binary logistic regression modelling (Table 4) was performed using the prespecified variables of age, gender, BMI, hernia size (measured as intrathoracic stomach percentage), CCI excluding age, strangulation, emergency presentation, hernia type, and intraoperative mesh use (including pledgets).

**Table 4:** Multivariate regression analysis for outcomes of patients undergoing LHHRaF between 2014 and 2020 at three public tertiary centres and three private hospitals in Melbourne, Australia

Variable	Post-operative morbidity			Comprehensive complication index score			Post-operative length of stay (days)		
	*P* value	Exp (B)	95% CI	*P* value	B	95% CI	*P* value	B	95% CI
Age	0.216	1.33	0.85–2.08	0.915	− 0.07	− 2.23 to 8.70	< 0.001	0.06	0.03–0.09
Gender (male)	0.286	1.42	0.75–2.70	0.310	− 0.85	− 2.49 to 0.80	0.235	− 0.45	− 1.12 to 0.30
BMI	0.563	1.02	0.96–1.09	0.455	− 0.06	−0.23 to 0.10	0.306	0.04	− 0.04 to 0.11
Intrathoracic stomach percentage	0.026	1.01	1.00–1.02	0.023	0.04	0.01 to 0.07	0.420	0.01	− 0.01 to 0.02
CCI (excluding age)	0.536	0.90	0.65–1.25	0.249	0.50	− 0.35 to 1.35	0.280	0.21	− 0.20 to 0.60
Strangulation	0.806	1.14	0.41–3.14	0.926	0.13	− 2.76 to 1.25	0.735	0.22	− 1.07 to 1.51
Emergency presentation	0.988	0.99	0.29–3.35	0.567	1.00	− 2.43 to 4.44	0.003	2.40	0.85–3.95
Type III/IV hernia	0.997	1.00	0.44–2.25	0.461	0.75	− 2.76 to 1.26	0.844	− 0.09	− 0.99 to 0.81
Mesh use (including pledgets)	0.723	1.16	0.52–2.60	0.751	0.31	− 1.61 to 2.24	0.696	− 0.17	− 1.03 to 0.69

Variable	Operative complications			Intraoperative blood loss (mL)			Operative duration (min)		
	*P* value	Exp (B)	95% CI	*P* value	B	95% CI	*P* value	B	95% CI
Age	0.754	0.84	0.28–2.51	0.140	0.37	− 0.12 to 0.86	0.012	0.60	0.13–1.07
Gender (male)	0.292	0.36	0.05–2.41	0.069	11.24	− 0.89 to 23.37	0.092	21.52	− 1.06 to 33.19
BMI	0.196	0.88	0.73–1.06	0.004	1.73	0.55–2.91	< 0.001	3.38	2.24–4.52
Intrathoracic stomach percentage	0.014	1.05	1.01–1.09	0.009	0.32	0.08–0.55	< 0.001	0.79	0.57–1.02
CCI (excluding age)	0.929	1.03	0.52–2.03	0.281	− 3.33	− 9.39 to 2.77	0.914	− 0.32	− 6.21 to 5.57
Strangulation	0.920	0.89	0.73–1.07	0.132	15.49	− 4.69 to 35.66	0.304	10.22	− 9.30 to 29.75
Emergency presentation	0.280	3.46	0.36–32.74	0.543	− 7.63	− 32.27 to 17.00	0.600	− 6.32	− 30.00 to 17.36
Type III/IV hernia	0.219	0.22	0.02–2.45	0.045	14.35	1.35–29.04	0.005	20.10	6.19–34.01
Mesh use (including pledgets)	0.465	0.47	0.06–3.78	0.839	1.42	− 12.32 to 15.16	0.577	6.73	− 9.49 to 17.01

Variable	Failure to improve reflux at 12-month post-procedure (DeMeester Score)			PPI requirement at 12-month post-procedure			Anatomical recurrence at 12-month post-procedure		
	*P* value	Exp (B)	95% CI	*P* value	B	95% CI	*P* value	B	95% CI
Age	0.343	1.34	0.69–1.48	0.875	1.06	0.53–2.10	0.188	0.93	0.84–1.04
Gender (male)	0.645	1.21	0.54–2.69	0.762	1.15	0.48–2.75	0.594	0.47	0.03–7.62
BMI	0.954	1.00	0.91–1.09	0.103	1.08	0.99–1.18	0.503	1.32	0.97–1.80
Intrathoracic stomach percentage	0.008	1.02	1.01–1.03	0.047	1.02	1.00–1.03	0.185	1.03	0.98–1.09
CCI (excluding age)	0.942	1.01	0.69–1.48	0.964	1.01	0.66–1.55	0.503	1.58	0.42–5.98
Strangulation	0.430	0.61	0.18–2.07	0.367	1.79	0.51–6.32	0.706	1.95	0.06–62.71
Emergency presentation	0.803	1.18	0.32–4.34	0.848	1.15	0.28–4.64	0.130	13.93	0.32–4.34
Type III/IV hernia	0.855	0.90	0.29–2.80	0.384	0.61	0.20–1.89	0.074	0.03	0.00–1.41
Mesh use (including pledgets)	0.854	1.10	0.38–3.18	0.204	2.01	0.67–6.58	0.901	0.82	0.20–3.11

Intrathoracic stomach percentage was shown to be predictive of post-operative morbidity (*P* = 0.026, OR 1.01, 95% CI 1.00–1.02), higher comprehensive complication index (*P* = 0.023, B 0.04, 95% CI 0.01–0.07), and operative complications (*P* = 0.014, OR 1.05, 95% CI 1.01–1.09). Each percentage point increase of intrathoracic stomach increased the odds of a post-operative complication by one percent and the odds of an operative complication by five percent. At 12 months, increasing intrathoracic stomach percentage was predictive of failure to improve reflux as assessed by DeMeester Symptom score (*P* = 0.008, OR 1.02 95% CI 1.01–1.03) and a persistent PPI requirement (*P* = 0.047, OR 1.02 95% CI 1.00–1.03) in this model. Three patients were excluded from PPI analysis at 12-month follow-up due to taking PPI therapy for non-reflux indications—in all three cases post-gastric or duodenal haemorrhage.

Intraoperative blood loss and operative duration were predicted by BMI (*P* = 0.004 and < 0.001, respectively), Type III/IV hernias (*P* = 0.045 and *P* = 0.005, respectively) and intrathoracic stomach percentage (*P* = 0.009 and *P* < 0.001, respectively). Post-operative length of stay was predicted by age (*P* < 0.001) and emergency presentation (*P* = 0.003). There were no significant predictors of anatomical recurrence at 12 months.

## Discussion

In this large, prospectively maintained multicentre series it was found that percentage of intrathoracic stomach was predictive of operative and post-operative morbidity.

Previous multivariate analyses for morbidity following hiatus hernia repair have identified other predictive factors. These include age [24, 41, 42], gender [24], emergency presentation [15, 24], ASA [41, 42], CCI domains of congestive cardiac failure and pulmonary disease [24], frailty [15], weight loss [41], gastrostomy [41] and gastropexy [42]. Most of these studies did not include hernia size as a variable and those that did measure this variable [24, 43] did not assess them against outcomes or include them in their regression models. As such, we believe that our multivariate findings are novel, as intrathoracic stomach percentage has not previously been compared against outcomes as a continuous quantitative variable. In addition, this study represents the first investigation of hernia size measured as percentage of intrathoracic stomach against outcomes whilst adjusting for potential confounders using multivariate regression modelling.

In this series, increasing percentage of intrathoracic stomach was also found to be the sole predictor of reflux symptom outcomes at 12 months, specifically failure to improve reflux as assessed by DeMeester Symptom Score and a persistent PPI requirement.

The relationship between the percentage of intrathoracic stomach and outcomes may be explained by many mechanisms. Larger hiatus hernias are usually a greater technical challenge to repair [1] due to their larger intrathoracic involvement and larger hiatal defects [12]. Repair of larger defects may lead to greater tension across the repair, which may account for the reduced efficacy of these repairs over time, as demonstrated by an increased PPI reliance and failure to resolve reflux symptoms at 12 months in those with greater percentages of intrathoracic stomach. Recurrence may also be related to this phenomenon. This was not demonstrated in our results; however, giant hiatus hernias have been previously significantly associated with recurrence [44], along with other factors, including age and obesity [3, 4].

Increased surgical trauma, which may manifest as intraoperative blood loss and operative duration during surgical repair, may contribute to post-operative morbidity. Previous meta-analysis evidence has shown longer operative duration to be a significant predictor of post-operative morbidity, with complication rates doubling in procedures longer than 2 h and increasing in a stepwise fashion for every 30 min of operative time [45]. Duration of anaesthesia has also been demonstrated as a significant predictor of overall post-operative morbidity [46] and respiratory complications after abdominal surgery [47]. Finally, intraoperative blood loss requiring transfusion has been associated with pulmonary, septic wound, and thromboembolic complications in non-cardiac surgery [48]. In our model BMI, high-grade hiatal hernias (Type III and IV) and percentage of intrathoracic stomach were all predictive of increased intraoperative blood loss and prolonged operative duration.

CT, barium swallow and manometry were approximately equivalent (7–8% mean variation) and endoscopy was the least accurate, accounting for the vast majority of cases with the greatest discrepancies. Disease progression between size estimation and repair and natural sliding hernia movements may account for some difference but would be present across all methods. Endoscopically we hypothesise that it is most difficult to estimate size due to the internal view appreciated and the ability to directly influence the size of the hernia during the procedure (e.g. Endoscopic hernia reduction).

These findings demonstrate that percentage of intrathoracic stomach represents a novel and important means of prognosticating post-operative morbidity and repair efficacy at relieving reflux symptoms. It also suggests that this may be accurately estimated radiologically, laparoscopically, manometrically or less accurately endoscopically preoperatively, with pre-operative and intraoperative measurements displaying a high degree of correlation. Percentage of intrathoracic stomach represents a simple to measure pre-operative predictor to assist in patient counselling prior to surgery. It may also be that larger hiatus hernias should undergo closer post-operative surveillance due to potentially poorer symptom resolution and higher recurrence rates.

Limitations in this study include potential selection bias due to its retrospective nature and in particular the difficulty in precisely measuring the percentage of intrathoracic stomach. The small number of collaborating high-volume oesophagogastric surgeons and consistency of operative technique may somewhat reduce this limitation. The 12- and 24-month follow-up rates were limited; however, our results are consistent with follow-up rates seen in other large studies investigating reflux surgery, with follow-up rates reported in a broad range between 39 and 91% [49, 50]. Additional loss to follow-up in this study is hypothesised to be related to a large proportion of rural patients, with telehealth yet to be largely adopted in Australia during the study period. Benign conditions have also been shown to be associated with clinic non-attendance in other cohorts [51]. These factors pose the possibility of introducing selection bias [52]. There was however no significant difference in mean intrathoracic stomach percentage at 12 months in those who attended follow-up compared to those who did not, which would somewhat limit the effect of this selection bias in relation to our findings. Despite this it presents a high-volume, multicentre, multi-surgeon study with a thorough multivariate model and as such, generalisable results.

## Conclusion

In a multivariate regression model of patients undergoing laparoscopic hiatus hernia repair and fundoplication, percentage of intrathoracic was predictive of operative and post-operative morbidity, failure to improve reflux and a persistent PPI requirement. BMI, high hernia grade and intrathoracic stomach percentage predicted operative duration and blood loss. Age and emergency presentation predicted post-operative length of stay.
